# The Story of the Discovery of the Circulation

**Published:** 1906-08

**Authors:** Roswell Park

**Affiliations:** Buffalo, N. Y.


### The Story of the Discovery of the Circulation.

**A Study of the Times and Labors of William Harvey.**

**Being the Doctorate Address Delivered at the Annual Commencement of the Medical Department of the University of Chicago, (Rush Medical College), June 13, 1906.**

By ROSWELL PARK, M. D., LL. D., Buffalo, N. Y.

HISTORY in general is but a record of the succession of great events or epochs which have moulded the world's affairs. That which is of the greatest import in the life of the individual may count for little in the lives of his contemporaries, and yet it must be said that in the events of today there has occurred a great epoch in the life of each of you, presumably the most important as yet in your personal records. This day is then in your personal histories one of the greatest importance. It is desirable, then, that your lives be so moulded and influenced by it that you may long hence look back to it and recall its significance.

I do not know what advice I can give you which will be more fruitful of results, than that among your studies you include that of the lives of the great men who have moulded destiny and made the world's history. Their lives were modified by little things, as have been and will be yours, and yet out of small matters grew for them and for us some of the most far reaching effects. Select the really great men who you best happen to know and analyze their characters that you may appreciate how they have become great; while if they have, as all great men have, traits of smallness, study even wherein they are small, and how such faults may be avoided.

History runs as does a fairly steady stream, save that every now and then some event abruptly diverts its course or influences its current. It has been so, for instance, with the history of medicine. For the first sixteen hundred years of the Christian era men engaged in the crude practices of our profession, utterly ignorant of the course of the blood, as well as of its purposes. Then appeared upon the scene a man who did his own thinking, who was willing to free himself from the shackles of the past, to observe nature and to reason therefrom. In this way came suddenly upon the world, as it were, an appreciation of the Circulation of the Blood, than which perhaps no event in medical history has been of greater importance or reflected more credit upon its demonstrator.

It is my purpose, then, today to try to tell you, in a semipopular way, how William Harvey came to make this great discovery, as well as to give you some idea of the difficulties under which he worked, and of the men and influences that surrounded him, believing that rather than spend a half hour in humorous platitudes which may provoke a smile but which are quickly forgotten, it is much better to try to implant something which may linger a while in your memories, and sufficiently impress you with the value of observation and inductive reasoning, since if you become thus fully impressed you will be spared in the future many sad errors of speech and even of thought.

Before telling the story of Harvey's life and work let us study for a few moments the general condition of affairs in Europe, in order that we may better understand the men whose influence surrounded him, as well as the spirit of the times and men's habits of thought.

Among the monarchs reigning in various parts of Europe during Harvey's time there were, for instance, in that part of the Empire of the West which was called Germany, Rudolph II, Matthias and Ferdinand. In Sweden reigned Kings Sigismund, Charles IX, the great monarch Gustavus Adolphus, and Queen Christine. In Prussia the throne had been occupied by Joachim, George William and Frederick William, as electors, this being before the days of the Prussian kings. In Russia the Czars Boris Godunow, Michael Theodore and Alexis had occupied the throne.

France had but recently passed through the inhuman butchery of the massacre of St. Bartholomew and its accompanying persecution of the Huguenots, under Charles IX, who expressed the hope that not a single Huguenot would be left alive to reproach him with the deed, but who died himself soon after the massacre, which is said to have caused him bitter remorse. Charles had been succeeded by his brother Henry III, a weak, fickle and vicious monarch, whose weakness caused him to be embroiled in civil strife, which was only concluded by his own assassination at the hands of a Dominican friar. Then came Henry IV, he of Navarre, afterwards surnamed The Great, who fought the famous battle of Ivry in 1590, and who reigned for twenty-one years, the greatest and most popular sovereign who ever occupied the throne of France. Notwithstanding his noble qualities he did not succeed in preserving his court from many of the contaminations of the age, and in his reign it is said that no less than 4000 French gentlemen were killed in duels, chiefly arising out of amorous quarrels. He was succeeded by Louis XIII, who was still on the throne when Harvey died.

In Harvey's own country James I was occupying the throne when Harvey appeared upon the scene. He was that royal pedant whom the Duke of Sully pronounced "the wisest fool in Europe." After his death, and when Charles I ascended the throne during his twenty-fifth year, in 1625, Harvey was preparing to publish his great work. It was this Charles I who retained as a favorite the worthless scoundrel Buckingham, whose misconduct in Spain prevented the proposed marriage of the king with the Spanish Infanta and brought about the Civil War. It was because of the cost of this war, and of the king's disputes with Parliament regarding the matter, that England was rent between the conflicts of the Cavaliers and the Roundheads, two of the consequences of this intestine strife being the execution of the Earl of Strafford and of Archbishop Laud. The troubles thus engendered finally cost the life of the king himself, who was beheaded in 1649. Harvey even lived to see the first half of the short tenure of office of Cromwell as the Great Protector, and was perhaps fortunate in dying before began the reign of that odious profligate Charles II.

It is worth while to enquire for a moment what was doing on this side of the ocean at this period which we have now under consideration. In 1607 Virginia was settled by the English, in 1614 New York by the Dutch, in 1620 Massachusetts and, three years later, New Hampshire, by the English Puritans; in 1624 New Jersey by the Dutch, in 1627 Delaware by Swedes and Finns, in 1630 Maine by the English, in 1634 Maryland by Irish Catholics, in 1635 Connecticut by English Puritans. Thus it will be seen that the active period of Harvey's life was synchronous with the beginnings of our colonial activities. Very little knowledge of what was going on in the then world of science was brought to this country at this period of its existence, however, and it was many years before in these colonies there were any exhibitions of scientific interest save in extremely scattered and sporadic cases.

Among Harvey's literary associates were a number of celebrated English poets, for example,—Marlowe (1593), Spenser (1598), Beaumont (1615), Shakespeare (1615), Herbert (1635), Ben Jonson (1637), Massinger (1639). Lord Bacon died a year or two after the appearance of Harvey's book, while Baron Napier, the inventor of logarithms, had passed away. His contemporaries in Italy, where he had studied, included Tasso (1595) and Galileo (1645). Rubens had died in 1640, Michael Angelo in 1564 and Titian in 1576. In France, Calvin, the practical murderer of Servetus, had passed away in 1564, Beza died in 1605, Descartes in 1650, Pascal in 1662 and Gassendi in 1655. Portugal had produced but one great figure in the 16th century, namely Camoens, who died in 1579. In Spain, Loyola, the ascetic and fanatic founder of the Jesuits, had joined the great majority in 1556; but Cervantes did not die until 1616, Lope de Vega in 1635, Velasquez in 1660 and Calderon in 1667.

In Germany some great figures had but recently disappeared. Paracelsus died in 1541, Copernicus in 1543, Luther in 1546, Hans Holbein in 1554, and Melancthon in 1560. Mercator, who introduced a new method of cartography, died in 1594, Tycho Brahe in 1601, Keppler in 1631, Van Dyck in 1641, Grotius, the great scholar, in 1645, Rembrandt in 1668 and Spinoza in 1677.

In philosophy, scepticism was the prevailing doctrine in the time of Harvey. It had been founded a hundred years previously by Montaigne, and continued by Charron, the chaplain of Queen Margaret of Navarre, who died in 1603, and who declared all religion to be opposed to human reason;— a remarkable attitude for a chaplain to assume. Opposed to the scepticism of Harvey's day was the mystic, Cabalistic or supernatural philosophy especially represented by Böhme, a peasant shoemaker, uneducated and yet wonderfully gifted. He had been the philosophical colleague of that great Meistersinger, Hans Sachs. Later philosophers and thinkers, yet belonging to Harvey's time, were Pascal, the great Jansenist, who discovered the variations of atmospheric pressure at different levels, and Malebranche, who figures prominently in the history of philosophy.

Descartes, who died in 1650, held the pineal gland to be the seat of the soul. He was the discoverer of the laws of refraction of light and furnished the explanation for the rainbow. He attained greatest eminence in mathematics, physics and philosophy, and was one of the inventors of modern algebra. One of his greatest opponents was that noble Jew, Spinoza, whose colleagues had expelled him from the Sanhedrim to the sound of the trombone.

The Italian Dominican Campanella, who died in 1639, considered the foundation of knowledge to be supernatural revelation and its perception by the senses. In spite of these views he came before The Inquisition on a charge of heresy and of cooperation with the Turks, was tortured by the rack and imprisoned for thirty years.

The mystic or Cabalistic notions of Harvey's day have just been mentioned. Under them we may recognize many degenerate products and amalgamations of the real doctrines of Paracelsus. The doctrines of the Rosicrucians, as well as of Zoroaster and the Cabala, were revived and made to do strange work. There was, for instance, that Sir Kenelm Digby, who died in 1605, a King's chamberlain, who posed among the English as a so-called Rosicrucian. It was he who suggested the famous *"sympathetic powder,"* which was to be applied to the weapon by which a wound had been inflicted, after which the *weapon* was anointed and dressed two or three times a day, while the wound itself was carefully bound up with dressings and left alone for a week. This was perhaps much the better course, but it will show what strange notions prevailed in those days.

What it meant to run counter to ecclesiastical policy and theological dogma appears not only in such tragedies as terminated the lives of Bruno and many other martyrs to science, but in such facts as these; for instance, when in 1624, just when Harvey was preparing to publish his work, some young chemists in Paris, seeing the benefit of the experimental method, broke away from Aristotle and the canons of theological reasoning, the faculty of theology appealed to the Parliament of Paris, which latter prohibited all such researches, under the severest penalties.

This was the time too when such exhibitions as the following were altogether too frequent;—One Quaresmio, of Lodi, came out with a ponderous work entitled "A Historical, Theological and Moral Explanation of the Holy Land," in which he devoted great space to the question of The Dead Sea and the salt pillar supposed to represent Lot's wife, dividing a long chapter upon the subject into three parts, dealing with the method and the locality of this transformation and the question of the existence at that time of her saline remains. Thus, with his peculiar powers of reasoning, he was able to decide the exact point where the saline change took place, and finally showed that the statue was still in existence.

Lord Bacon was also an older contemporary of Harvey, having been born in 1561 and dying in 1626, shortly after the appearance of Harvey's great work. His services to analytic science need no description here, but it is worth while to remember that Harvey, like many others, must have come under his influence and have profited by his teachings in logic and analysis.

At about the time when Harvey made known his discovery Bacon was publishing his views of the laws of transmission and reflection of sound. Great man as he was, with a keen foresight into the value of the recent inventions of the compass, gunpowder and printing, he nevertheless was himself so narrow, in some respects, that he placed but little value upon the discovery of Copernicus. He, however, paved the way for one in some respects still greater, namely Isaac Newton, who, however, had scarcely attained man's stature when Harvey died.

How much we owe to the two great Bacons of history one cannot indicate in this short résumé. Roger Bacon (1214-1292) seems to have been the first great thinker along truly scientific lines. He was more than a mere chemist and, as White says, more than three centuries before Francis Bacon *advocated* the experimental method Roger Bacon had *practised* it, and in many directions. He did more than anyone else in the middle ages to direct thought into fruitful paths, and only now are we finding out how nearly he reached some of the principal doctrines of modern philosophy and chemistry. Most important of all, his methods were even greater than his results, and this at a time when "theological subtilizing" was the only passport to reputation for scholarship.

It was Avicenna, the Arabian, who perhaps first announced substantially the modern theory of geology, accounting for changes in the earth's surface by suggesting a stone-making force, but the presence of fossils in the rocks had been always a thorn in the sides of the theologians. It was Leonardo da Vinci, that versatile genius in science and art, who, previous to Harvey's generation, suggested true notions as to the origin of fossils, while, in Harvey's time, Bernard Palissy, another artist, vehemently contended for their correctness. Still, even at Harvey's death, neither geology nor paleontology had come anywhere near scientific accuracy.

The *Academia dei Lyncei,* so-called from its seal, which bore the image of a fox, was founded in Rome in 1603. In France The Academy of Science was not founded until 1665, in Germany The Society of Naturalists and Physicians in 1652, and the British Royal Society in 1665.

In matters of general interest it may be worth while to say that in architecture the general style of The Renaissance was changed for the more substantial Barocco, while the more formal and limited style of church music had given away to musical drama, i. e., opera, albeit in very crude form. The first newspaper had appeared at Antwerp in 1605, the first German paper being published in Frankfort in 1615, and The London Weekly News making its first appearance in 1620. Tobacco, which had been brought over by Raleigh in 1560, had come into quite general use, while coffee, tea and chocolate had gained in public esteem. When coffee was first introduced in England it sold for about $28 a pound. The first coffee house appears to have been established in Constantinople, in the middle of the 16th century, while the first coffee house in London was not opened until a century later.

The barbers still retained their ascendency, and the bath keepers had scarcely lost their position next to the barbers. It was not until Harvey had reached a ripe age that the barbers were required in Germany to pass an examination, in which they had to prove not only their knowledge but the legitimacy of their birth, and the fact that they had studied for three years and had worked for three years more as apprentices.

Anatomy was studied quite generally, sometimes upon human bodies. A dissecting room had been established in Dresden in 1617, in which stuffed bears, at that time a great rarity, were preserved with other curiosities. In 1623 Rolfink, at Jena, arranged for public dissection upon the bodies of all executed malefactors, delegates being present thereat from various other institutions. It is worth while to mention that in Frankfort, for instance, during the expiration of 65 years, but seven dissections were made, and that these were always accompanied by a celebration which lasted several days. Vienna did not possess a skeleton in 1668, and Strassburg did not have one until 1671. Yet it is of interest to remember that the anatomical plates, like those often published today, which are meant to be lifted off in layers, existed even at this period. On the other hand, botanical gardens and chemical laboratories existed in several of the universities,—in Strassburg, for instance, in 1619,—in Oxford in 1622.

Fabricius Hildanus, the father of German surgery, or, as he has been sometimes called, the Ambroise Paré, of Germany, was also a contemporary of Harvey's. His real name was Fabry and he was born in Hilden, but he latinized his name into that form usually adopted today.

Scultetus was another famous surgeon of the same period.

William Gilbert, 1540-1603, had been the talented physician of Queen Elizabeth, and was among the first to study the experimental method. With the appearance of his book upon the magnet, in 1600, began the science of electricity and magnetism. He was the first to teach the fact that the earth itself was a great magnet and he distinguished between magnetic and electric reactions. Later the great Dutch anatomist, Ruysch, afforded corroboration of Harvey's views by another method, when he invented and practised those beautiful minute injections of the vascular system which made him so famous, and who built up that great collection of specimens which Peter the Great bought for Russia at an expense of about $75,000.

Contemporary with Harvey also was Swammerdam, one of the most versatile men of his time, famous as naturalist, savant, physiologist, linguist and poet. It was during the fifteenth century that astronomy began to assume an importance and degree of accuracy never hitherto known. This was due very largely to the independence of thought and the researches of Copernicus, who was born in Cremona in 1477, and who studied medicine in Krakau and astronomy in Vienna. He lived to the age of 70 and was the real father of the heliocentric theory, now known as the Copernician system, which he substituted for the previous Ptolemaic theory, thus reversing the ancient idea that the sun circled about the earth. Copernicus demonstrated the phases of the moon, but his opponents claimed that if this doctrine were true Venus would exhibit the same phenomena; to which he replied that it was true, though he knew not what to say to these objections, but that God was good and would in time furnish answer to them. It was Galileo's crude telescope which, in Harvey's younger day, in 1611, furnished this answer and revealed the phases of Venus. To illustrate how the views of Copernicus were received we might add here that Martin Luther paid his compliment to him by declaring that Copernicus was a fool who wished to stand astronomy upon its head.

Copernicus was succeeded by Galileo, who was born in 1554 in Piza, and died 1642. He may be called the creator of dynamic astronomy and mechanics, as well as one of the most brilliant exponents of experimental and inductive reasoning. He was of noble birth and was, in fact, the torch bearer of physics at the period of The Renaissance. He gave up speculation and substituted for it the habit of observation, reaping a large harvest of surprising facts, any one of which might have immortalized him. He not only established the movements of the earth on its own axis as well as around the sun, which Copernicus had shown, but he discovered the weight of the atmosphere and first calculated the law of gravity. He and his successors were governed always by that aphorism which is today as true as ever: "Experience is deceptive and judgment difficult."

In 1615 when he was before The Inquisition at Rome, and when its theologians had examined statements extracted from his letters, they solemnly rendered their decision in these words: "The first proposition that the sun is the centre and does not revolve about the earth is foolish, absurd, false in theology and heretical, because expressly contrary to The Holy Scripture. The second proposition that the earth is not the centre, but revolves about the sun, is absurd, false in philosophy and, from a theological point of view, at least, opposed to the true faith." This for a pronunciamento from the *infallible* Church!

Galileo and Bruno have by some writers both been made to stand in an unpleasant light because of their recantation or shifting position before The Inquisition. Bruno was the greatest philosopher and sceptic of the latter part of the 16th century, and had outlined, withal somewhat vaguely, that which is now known as the nebular hypothesis. He was murdered by The Inquisition in 1600, and the views which he enunciated seem to have been buried with him, not to reappear until long after his sad fate had been consummated. He had, for instance, contended for the truths of the Copernican doctrine, but it was not until ten years after his martyrdom that Galileo proved it with his telescope. That both these great men yielded in some respects to the influences of The Inquisition and renounced some of their scientific "heresies" is largely to be excused by the fact that they were both old, broken in health from the sufferings which they had endured, as well as from their disappointments, and that they had been, under these circumstances, handed over to that Inquisition which knew no mercy. Galileo could well remember the *auto da fê* in the Piazza dei Fiore, in Rome, the scene of Bruno's martyrdom, as well as the tragic end of many another who had dared to have the courage of his convictions. Let us, then, not judge him harshly, but be grateful even that the enormous power of The Inquisition did not and could not suppress the truth.

Galileo's discovery of the satellites of Jupiter, the rings of Saturn, his experiments with the pendulum, his construction of the telescope, as well as of the thermometer, and many other deeds, have stamped him as one of the great figures in the history of progress and science. It is most interesting to note that this contemporary of Harvey's like himself, was given to induction obtained from experimental studies. Another great astronomical light of Harvey's time was Keppler, who was driven from one place to another by religious fanaticism, until he ended his life in 1630. It was he who formulated the great principle which underlies the motions of the planets, and who gave to the world his so-called "laws," which so materially advanced the science of astronomy. It was he who really discovered that comet which was later given Halley's name, whose periodic return he first foretold.

Such was the spirit of the times in which Harvey lived, and such the influences which surrounded his teachers before him and himself in turn. It makes a long preface to a consideration of what Harvey himself accomplished, but it is not without its interest because men and their works must be judged largely by their environment. Now, to speak more particularly of Harvey himself, and what was known of the circulation when he undertook his investigations.

The liver had been considered, from time immemorial, as the principal factor in the production and movement of the blood. The ancients supposed that here the veins took their origin and that through them the blood flowed to all parts of the body, returning to its source by an undulating movement or series of alternate waves. The arteries had been supposed to contain only vital spirits, whose great reservoir was the heart, although Erasistratus had admitted that in certain cases blood might escape into the arterial channels. Later Galen showed that the arteries always contained blood, and he knew that blood was poured into the right side of the heart by the great veins, but believed that only a little of it passed from the right ventricle into the lungs, the greater part of it passing through hypothetical pores in the septum and thus into the left ventricle. This opinion, like Galen's in other respects, remained unchanged until the middle of the 16th century. It was also known that valves existed within the veins, and that if an artery were tied on a living animal blood would cease to flow and pulsation be checked below the ligature, while if a vein were tied it shrunk above the ligature and became distended below.

Three men before Harvey's time came very near to discovering the secret that made him famous; in fact, they made such advances on what was already known that history should accord them a distinguished place. One was *Columbus*, who was born at Cremona in 1490, and died in 1559. He was first a pupil and prosector and then a friend of Vesalius, the great anatomist. Later he succeeded him at The University of Padua and unfortunately, after gaining his position, ungratefully turned upon his old teacher. He was, however, for his day a good anatomist and especially a good osteologist. It was he who first demonstrated experimentally that blood passes through the lungs into the pulmonary veins and that the latter connect with the left ventricle. He thus practically established the fact of the lesser circulation. He suffered, however, as did Servetus, from the prevailing notion that spirits and blood were mixed together. From Padua Columbus went to Pisa, and then to Rome. He wrote with elegance and correctness of style and even described the vessels which penetrate the bone cells, the ossicles of the ear, the minute anatomy of the teeth, the ventricles of the larynx, as well as those valves which prevent the return of blood from the lungs to the heart. In fact, he narrowly missed the significance of the actual facts of the case, simply failing in his final analysis and assembling of those facts which he had already demonstrated.

*Cesalpinus,* who lived a little later, came still nearer the mark, having accepted the teachings of Columbus regarding the course of the blood through the lungs. He added that the ultimate arterial branches connect with those of the veins, and he taught that blood and vital spirits, from which the ancients could never separate themselves, passed from the arteries into the veins during sleep, as was demonstrated by the swelling of the veins and the diminution of the pulse at that time.

A little later came *Michael Servetus,* who figures principally in history as a theologian and a victim of theologians, since he perished a martyr to Calvin's jealousy. He was, in effect, a wisely and widely educated man who did a great deal for science, one of the offences attributed to him being an edition of Ptolemy's geography, in which Judea was described as a barren and inhospitable land instead of one "flowing with milk and honey." This simple statement of a geographical fact was made a tremendous weapon of offence by Calvin, who replied that even if Servetus had only quoted from Ptolemy and, although there were ample geographical proofs, it nevertheless "unnecessarily inculpated Moses and grievously outraged The Holy Ghost." Servetus dared to deny the passage of the blood through the septum of the heart, and contended that that which comes into the right side was distributed to the lung and returned to the left ventricle. He published his views, however, in a religious treatise on Errors concerning The Trinity, a most unfortunate place in which to inject such an important fact, since it gave his enemies a still greater opportunity to vent and ventilate their spleen. Had he been able to leave out that notion of vital spirits, which prevailed with all his predecessors, he might actually have made the great discovery left for Harvey to enunciate. I have not been able to refer to original documents in this matter, but it is claimed by some that his description of the circulation was contained in another religious work concerning the Restitution of Christianity, which was printed in Nuremburg in 1790.

Such was the actual state of knowledge concerning the movements of the blood and the functions of the heart when Harvey published his great work. It behooves us now to proceed with a short account of Harvey's own life and researches.

*William Harvey* was born at Folkstone on the first of April, 1578. He was the eldest son of a prosperous merchant who raised a large family and who occupied the highest positions of honor in his own town. The son William was born to his second wife, by whom he had seven sons and two daughters. All of these children were helped to remunerative or honorable positions. They became merchants or politicians or secured prominence in some way, but William was the only one to study medicine. He was sent to the King's school at Canterbury, in 1588, and he was admitted at Caius in Cambridge in 1593, where he graduated in arts in 1597. The following year he went to Padua, which then had one of the greatest medical schools of the time, and he obtained his medical diploma in 1602, when twenty-four years of age. Returning to England he received a doctor's degree at Cambridge, and shortly afterward married a daughter of a London physician and entered upon the practice of medicine in London.

In the great city his practice as a physician seems to have been from the outset successful, and his knowledge and ability procured him various valuable appointments. He was made a Fellow of The College of Physicians in 1607. This Royal College of Physicians was given a grant of incorporation by Henry VIII in 1518, at the intercession of Chambers, Linacre and Ferdinand Victoria, the King's Physicians, it being under the patronage of Cardinal Woolsey. The first meetings were held at Linacre's house which he bequeathed to the corporation at his death. Until this College was founded practitioners of medicine were licensed to practise by the Bishop of London or by the Dean of St. Paul's.

A few years later Harvey was appointed Physician-Extraordinary to King James I, and later yet, after the publication of his great treatise and its dedication to the King, he was made Physician-in-Ordinary to Charles I, whom he attended during the Civil Wars.

It must have been about 1615 when Harvey first began expounding his views on the circulation of the blood, during lectures which were delivered at The College of Physicians, but it was not until thirteen years later, i. e., in 1628, that his great work DE MOTU CORDIS was published in Latin, as was customary among scholars, and at Frankfort-on-the-Main, since that was the great center of the book publishing trade at that time.

The treatise was dedicated to King Charles I, in a manner which to us would seem servile, and yet which was according to a custom followed by nearly all of the scholars of the day, who desired to attract not only the attention of royalty, but, in most instances, their benevolent assistance. It is worth while to quote at this point the first sentence or two of his dedication:

"To the<br>

Most Serene and Invincible<br>

CHARLES,<br>

of Great Britain, France and Ireland,<br>

KING: DEFENDER of the FAITH,<br>

Most Serene King,

"The heart of animals is the basis of their life, the principle of the whole, the Sun of their Microcosm, that upon which all movement depends, from which all strength proceeds. The King in like manner is the basis of his Kingdom, the Sun of his World, the heart of the Commonwealth, whence all power derives, all grace appears. What I have here written of the movements of the heart I am the more emboldened to present to your Majesty, according to the Custom of the present age, because nearly all things human are done after human examples and many things in the King are after the pattern of the heart."

The dedication was followed by a Proemium which one may hardly read today without emotion. In it he sets forth the mystery that has surrounded the subject of the motion and function of the heart, as well as the attendant difficulties of the subject, speaking of his own early despair that he would ever be able to clear up the subject. He even said that at one time he found the matter so beset with difficulties that he was inclined to agree with Fracastorius "that the movements of the heart and their purpose could be comprehended by God alone." Only later was this despair dispelled by a suggestion when, as he says: "I began to think whether there might not be a movement in a circle" and when thus the truth dawned fully upon him.

We shall have to speak later of the opposition provoked by the appearance of this work and its almost general rejection. It is perhaps, however, but just to those who disputed Harvey's discoveries to recall that no complete and actual demonstration of the actual circulation was possible at that time, nor for many years after, and until the introduction of the microscope, the common magnifying glass of that day being the only lens in use. It remained for Malpighi to demonstrate the blood actually in circulation in the lung of a frog some three or four years after Harvey's death, in 1657. But Harvey lived long enough to see his views gain general acceptance, and though at first, and as the result of the opposition provoked by his publication, his practice fell off mightily, he later regained his professional position and rose to the highest eminence, being elected in 1654 to the Presidency of the College of Physicians. To this institution he proved a great benefactor, making considerable additions to the building after its destruction in The Great Fire of 1666 and its subsequent restoration. He also left a certain sum of money as a foundation for an annual oration, to be delivered in commemoration of those who had been great benefactors of the College. This oration is still regularly delivered on St. Luke's Day, i. e., the 18th of October, and is ordinarily known as the Harveian oration. In these orations more or less reference to Harvey's work and influence is always made.

This great man passed away on the 3d of June, 1657, within ten months of his eightieth birthday, thus affording a brilliant exception to the list of men who have rendered great service to the world and not lived long enough to see it appreciated.

As one reads Harvey's own words, the wonder ever grows that it should have remained for him, after the lapse of so many centuries, to not only call attention to what had been said by Galen but apparently forgotten by his successors, namely, that "the arteries contained blood and nothing but blood, and, consequently, neither spirits nor air, as may be readily gathered from experiments and reasonings," which he elsewhere furnishes. He furthermore shows how Galen demonstrated this by applying two ligatures upon an exposed artery at some distance from each other, and then opening the vessel itself in which nothing but blood could be found. He calls attention also to the result of ligation of one of the large vessels of an extremity, the inevitable result being just what we today know it must be, and the procedure terminating with gangrene of the limb.

Not long before Harvey's own publication, Fabricius, he of Aquapendente, had published a work on respiration, stating that, as the pulsation of the heart and arteries was insufficient for the ventilation and refrigeration of the blood, therefore were the lungs fashioned to surround the heart. Harvey showed how the arterial pulse and respiration could not serve the same ends, combating the view generally held, that if the arteries were filled with air, a larger quantity of air penetrating when the pulse is large and full, it must come to pass that if one plunge into a bath of water or of oil when the pulse is strong and full it should forthwith become either smaller or much slower, since the surrounding fluid would render it either difficult or impossible for air to penetrate. He also called attention to the inconsistencies between this view and the arrangement of the prenatal circulation; also to the fact that marine animals, living in the depths of the sea, could under no circumstances take in or emit air by the movements of their arteries and beneath the infinite mass of waters, inasmuch as "to say that they absorb the air that is present in the water and emit their fumes into this medium, were to utter something very like a figment;" furthermore "when the windpipe is divided, air enters and returns through the wound by two opposite movements, but when an artery is divided blood escapes in one continuous stream and no air passes."

Discussing further the views which he stigmatized as so incongruous and mutually subversive that every one of them is justly brought under suspicion, he reverts again to the statements of Galen, calling attention to the fact that from a single divided artery the whole of the blood of the body may be withdrawn in the course of half an hour or less, and the inevitable consequences of such an act; also that when an artery is opened the blood is emptied with force and in jets, and that the impulse corresponds with that of the heart; again that in an aneurism the pulsation is the same as in other arteries, appealing for corroboration in this matter to the recent statements of Riolan, who later became his avowed enemy. Harvey also called attention to the fact that while ordinarily there was a seemingly fixed relation between respiration and pulse-rate, this might vary very much under certain circumstances, showing that respiration and circulation were two totally different processes. Harvey utilized also the results of his researches in comparative anatomy and physiology, for early in his work he calls attention to the fact that every animal which is unfurnished with lungs lacks a right ventricle.

In his Proemium he then proceeds to ask certain very pertinent questions which can only be briefly summarized in this place. He asks: First, why, inasmuch as the structure of both ventricles is practically identical, it should be imagined that their uses are different, and why, if tricuspid valves are placed at the entrance into the right ventricle and prove obstacles to the return of blood into the vena cava, and if similar valves are situated at the commencement of the pulmonary artery, preventing return of blood into the ventricle, then why, when similar valves are found in connection with the other side of the heart, should we deny that they are there for the same purpose of preventing "here the egress" and "there the regurgitation of the blood?"

Secondly, he asks why, in view of the similarity of these structures, it should be said that things are arranged in the left ventricle for the egress and regress of spirits, and in the right ventricle for those of blood?

Thirdly, he enquires why, when one notes the resemblance between the passages and vessels connected with the opposite sides of the heart, one should regard one side as destined to a private purpose, namely, that of nourishing the lungs, the other to a more public function? Furthermore, he enquires, since the lungs are so near, and in continual movement, and the vessels supplying them of such dimensions, what can be the use of the pulse of the right ventricle, which he had often observed in the course of his experiments? He sums up his inability to accept the explanations previously offered with a phrase which reads rather strangely, even in original Latin: "Deus bone! Quomodo tricuspides impediunt aëris egressum, non sangiunis." i. e., "Good God! how should the mitral valves prevent the regurgitation of air and not of blood?"

He then takes up the views of those who have believed that the blood oozed through the septum of the heart from the right to the left side by certain secret pores, and to them he replied "By Hercules, no such pores can be demonstrated, nor, in fact, do any such exist." Again, "Besides, if the blood could permeate the substance of the septum, or could be emptied from the ventricles, what use were there for the coronary artery and vein, branches of which proceed to the septum itself, to supply it with nourishment?"

Further on in the treatise Harvey sets forth his motives for writing, stating how greatly unsettled had become his mind in that he did not know what he himself should conclude nor what to believe from others. He says: "I was not surprised that Laurentius should have written that the movements of the heart were as perplexing as the flux and reflux of Euripus had appeared to Aristotle." He apologizes for the crime, as some of his friends considered it, that he should dare to depart from the precepts and opinions of all anatomists. He acknowledged that he took the step all the more willingly, seeing that Fabricius, who had accurately and learnedly delineated almost every one of the several parts of animals in a special work, had left the heart entirely untouched.

Passing more directly to the actual work of the heart, he shows that not only are the ventricles contracted by virtue of the muscular structure of their own walls, but further that those fibers or bands, styled "Nerves" by Aristotle, that are so conspicuous in the ventricles of larger animals when they contract simultaneously, by an admirable adjustment, help to draw together all the internal surfaces as if with cords, thus expelling the charge of contained blood with force. Later on he says that if the pulmonary artery be opened, blood will be seen spurting forth from it, just as when any other artery is punctured, and that the same result follows division of the vessel which in fishes leads from the heart. He furnishes a very happy simile to prove that the pulses of the arteries are due to the impulses of the left ventricle by showing how, when one blows into a glove ali of its fingers will be found to have become distended at one and the same time. He quotes Aristotle, who made no distinction between veins and arteries, but said that the blood of all animals palpitates within their vessels and by the pulse is sent everywhere simultaneously, all of this depending upon the heart.

It is in Chapter Five of the treatise that he gives, probably for the first time, an accurate published account of just what transpires with one complete cycle of cardiac activity. The passage need not be quoted here, but deserves to be read by everyone interested in the subject, as who should not be? One sentence, however, is worth quotation or, at least, a summary, as follows: "But if the divine Galen will here allow, as in other places he does, that all the arteries of the body arise from the great artery, and that this takes its origin from the heart; that all the vessels naturally contain and carry blood; that the three semilunar valves situated at the orifice of the aorta prevent the return of the blood into the heart, and that they were here for some important purpose, —I do not see how he can deny that the great artery is the very vessel to carry the blood, when it has attained its highest triumph of perfection, from the heart for distribution to all parts of the body."

His Chapter Six deals with the course by which blood is carried from the right into the left ventricle, and here one must admire the large number of experimental demonstrations which Harvey had undertaken upon all classes of animals, for he speaks even of that which occurs in small insects, whose circulation he had studied so far as he could with the simple lens. Furthermore he described the prenatal circulation, omitting practically nothing of that which is taught today, showing that in embryos, while the lungs are yet in a state of inaction, both ventricles of the heart are employed, as if they were but one, for the transmission of blood. In concluding this chapter he again states briefly the course of the blood, and promises to show, first, that this may be so and, then, to prove that it really is so.

His Chapter Seven is devoted to showing how the blood passes through the substance of the lungs from the right ventricle and then on into the pulmonary vein and left ventricle. He alludes to the multitude of doubters as belonging, as the poet had said, to that race of men who, when they will, assent full readily, and when they will not, by no matter of means; who, when their assent is wanted, fear, and when it is not, fear not to give it. A little later on he says: "As there are some who admit nothing unless upon authority, let them learn that the truth I am contending for can be confirmed from Galen's own words, namely, that not only may the blood be transmitted from the pulmonary artery into the pulmonary veins and then into the left ventricle of the heart, but that this is effected by the ceaseless pulsation of the heart and the movements of the lungs in breathing." He then shows how Galen explained the uses of the valves and the necessity for their existence, as well as the universal mutual anastomosis of the arteries with the veins, and that the heart is incessantly receiving and expelling blood by and from its ventricles, for which purpose it is furnished with four sets of valves, two for escape and two for inlet and their regulation.

Harvey then noted a well-known clinical fact, that the more frequent or forcible the pulsations, the more speedily might the body be deprived of its blood during hemorrhage, and that it thus happens that in fainting fits and the like, when the heart beats more languidly, hemorrhages are diminished and arrested. The balance of the book is practically devoted to further demonstration and corroboration of statements already made. A study of this work of Harvey's illustrates how much respect even he and his contemporaries still showed for the authority of Galen. It shows still further how nearly Galen came to the actual truth concerning the circulation. Had the latter not adopted too many of the notions of his predecessors concerning the nature of the soul (Anima) and the spirits (Pneuma) of man, he might himself have anticipated Harvey by a thousand years, and by such announcement of a great truth have set forward physiology by an equal period. Independent and original as Harvey showed himself, he seems to have failed to get away from the notion of the vapors and spiritual nature of the blood which he had inherited from the writings of Galen and many others. Nevertheless he also alludes to this same blood as alimentive and nutritive. We must not forget, however, that this was years before Priestly's discovery of oxygen and that Harvey had, like others, no notion of the actual purpose of the lungs, believing that the purification and revivification of the blood was the office of the heart itself.

Along with its other intrinsic merits Harvey's book possesses a clear and logical arrangement, the author first disposing of the errors of antiquity, describing next the behavior of the heart in the living animal, showing its automatic pumplike structure, its alternate contractions and the other phenomena already alluded to, thus piling up facts one upon another in a manner which proved quite irresistible. The only thing that he missed was the ultimate connection between the veins and the arteries, i. e., the capillaries, which it remained for Malpighi to discover with the then new and novel microscope, which he did about 1657, showing the movements of the blood cells in the small vessels, and confirming the reality of that ultimate communication which had been held to exist. Malpighi discovered the blood corpuscles in 1665, but it remained for Leeuwenhoek of Delft, in 1690, by using an improved instrument to demonstrate to all observers the actual movements of the circulating blood in the living animal. One historian has said that with Harvey's overthrow of the old teachings regarding the importance of the liver and of the spirits in the heart fell the four fundamental humors and qualities, while Daremberg exclaims: "As in one of the days of the creation chaos disappeared and light was separated from darkness."

It remains now only to briefly consider how Harvey's great discovery was received. To quote the words of one writer: "So much care and circumspection in search for truth, so much modesty and firmness in its demonstration, so much clearness and method in the development of his ideas, should have prepossessed everyone in favor of the theory of Harvey; on the contrary, it caused a general stupefaction in the medical world and gave rise to great opposition.

During the quarter of a century which elapsed after Harvey's announcement there probably was not an anatomist nor physiologist of any prominence who did not take active part in the controversy engendered by it; even the philosopher Descartes was one of the first adherents of the doctrine of the circulation, which he corroborated by experiments of his own.

Two years after the appearance of Harvey's book appeared an attack, composed in fourteen days by one Primerose, a man of Scotch descent, born and educated in France, but practising at Hull, in which he pronounced the impossibilities of surpassing the ancients or improving on the work of Riolan, who already had written in opposition to Harvey, and who was the only one to whom the latter vouchsafed an answer. It was Riolan who procured a degree of the Faculty of Paris prohibiting the teaching of Harvey's doctrine. It was this same Riolan who combated with equal violence and obstinacy the other great discovery of the age, namely,—the circulation of the lymph.

One of the earliest and fiercest adversaries of Harvey's theory was Plempius, of Louvaine, who, however, gave way to the force of argument and who finally publicly and voluntarily passed over to the ranks of its defenders in 1652, becoming one of Harvey's most enthusiastic advocates.

Harvey's conduct through the controversy was always of the most dignified character; in fact, he rarely ventured to reply in any way to his adversaries, believing in the ultimate triumph of the truths which he had enunciated. His only noteworthy reply was one addressed to Riolan, then Professor in the Paris Faculty and one of the greatest anatomists of his age, to whose opinion great value was always attached. Even in debating or arguing against him, Harvey always spoke of him with great deference, calling him repeatedly The Prince of Science. Riolan was, however, never converted, though whether he held to his previous position from obstinacy, from excess of respect for the ancients, or from envy and jealousy of his contemporary, is not sure.

Another peculiar spectacle was afforded by one Parisunus, who died in 1643, a physician in Venice, who, like Harvey, had been a pupil of Fabricius of Aquapendente, who had been stigmatized by Riolan as an ignoramus in anatomy, but who joined with others in declaring that he had seen the heart beat when perfectly bloodless, and that no beating of the heart and no sounds were to be heard as Harvey had affirmed.

With the later and more minute studies into the structure and function of the heart we are not here concerned. The endeavor has been rather to place before you the sentiments, the knowledge and the habits of thought of the men of Harvey's time, with the briefest possible epitome of what they knew, or rather of how little they knew, to account for this later slavish adherence to authority by unwillingness to reason independently or to observe natural phenomena intelligently, still less to experiment with them. It is, then, rather the brief history of an epochal discovery than an effort to trace out its far-reaching consequences that I have endeavored to give.

Here must close an account which perhaps has been to you tedious, and yet which is really brief, of Harvey's life and labors. He lived to see his views generally accepted and to enjoy his own triumph, a pleasure not attained by many great inventors or discoverers. Lessons of great importance may be gathered from a more careful study of this great historical epoch, but they must be left to your own powers of reasoning rather than to what I may add here. I commend it to you as a fertile source of inspiration, and a line of research worthy of both admiration and imitation. Few men have rendered greater service to the world by the shedding of blood than did Harvey in his innocent and wonderful studies of its natural movement. Perhaps it might be said of him that he as the first man to show that "blood will tell." What he made it tell has been thus briefly told to you.

I know not how I may better close this account than by quoting the concluding words of his famous book, and especially repeating the lines which he has quoted from some Latin author whom I have not been able to identify. His paragraph and his quotation are as follows:

"Finally, if any use or benefit to this department of the republic of letters should accrue from my labors, it will, perhaps, be allowed that I have not lived idly, and, as the old man in the comedy says:

'For never yet hath anyone attained
To such perfection, but that time, and place,
And use, have brought addition to his knowledge;
Or made correction, or admonished him,
That he was ignorant of much which he
Had thought he knew; or led him to reject
What he had once esteemed of highest price.'"

